# Ischemic stroke in the setting of supratherapeutic International Normalized Ratio following coronavirus disease 2019 infection: a case report

**DOI:** 10.1186/s13256-023-03936-8

**Published:** 2023-05-31

**Authors:** Gokhan Demir, Rama Hommos, Sally Sami Al-Sirhan, Hashem Abu Serhan, Muhannad Haddadin, Umar Bin Rashid, Yamane Chawa

**Affiliations:** 1 Department of Internal Medicine, Hamad General Hospital, Doha, Qatar; 2 College of Medicine, Qatar University, Doha, Qatar; 3 Department of Ophthalmology, Hamad General Hospital, Doha, Qatar

**Keywords:** Stroke, SARS-CoV-19, Ischemia, Infarction, INR, Middle cerebral artery

## Abstract

**Background:**

SARS-CoV-19 infection is associated with an increased risk of thrombotic events. We present a case of acute middle cerebral artery ischemic stroke in a patient with SARS-CoV-19 infection despite being on warfarin with supratherapeutic INR (International Normalized Ratio).

**Case presentation:**

A 68-year-old Caucasian female with multiple comorbidities was admitted to the hospital with symptoms of upper respiratory tract infection. A rapid antigen test confirmed the diagnosis of COVID-19 pneumonia, and intravenous remdesivir was initiated. On the fifth day of admission, the patient experienced sudden onset confusion, slurred speech, left-sided hemiplegia, right-sided eye deviation, and left-sided facial droop. Imaging studies revealed an occlusion of the distal anterior M2 segment of the right middle cerebral artery, and an MRI of the brain confirmed an acute right MCA infarction. Notably, the patient was receiving warfarin therapy with a supratherapeutic INR of 3.2.

**Conclusions:**

This case report highlights the potential for thromboembolic events, including stroke, in patients with COVID-19 infection, even when receiving therapeutic anticoagulation therapy. Healthcare providers should be vigilant for signs of thrombosis in COVID-19 patients, particularly those with pre-existing risk factors. Further research is necessary to understand the pathophysiology and optimal management of thrombotic complications in COVID-19 patients.

**Supplementary Information:**

The online version contains supplementary material available at 10.1186/s13256-023-03936-8.

## Background

Thus far, acute ischemic stroke (AIS) has been reported to complicate 1%–5% of coronavirus disease 2019 (COVID-19) infections [1]. Severe COVID-19 infection is associated with a high risk of thrombosis, since patients exhibit all three components of Virchow’s triad, and in those who experience a profound systemic inflammatory response to COVID-19 infection, there may be an increased risk of prothrombotic events [2, 3].

There have been reports of an increased tendency to develop coagulopathy in SARS-CoV-2-infected patients [4], and there is evidence that the degree of coagulopathy is correlated with the severity of respiratory sickness [5]. The main thrombotic event is recognized as venous thromboembolism, but stroke is also identified, although this conclusion is debatable [6] due to a lack of high-quality evidence. The process of increased risk of ischemic stroke in COVID-19 patients is not well-established, although different studies have proposed several mechanisms, including cytokine storm [7].

Thromboembolism prophylaxis has been used since the beginning of the COVID-19 pandemic, but it has become rapidly clear that a routine prophylactic dose—usually of low molecular weight heparin (LMWH)—was often insufficient to prevent venous thrombosis in these patients [1]. The high incidence of thrombotic events, even with prophylactic dose anticoagulation, has led physicians to use intermediate or even therapeutic doses of anticoagulants [3].

This case report presents a unique and challenging scenario of a patient who was hospitalized with COVID-19 and later developed an acute ischemic stroke. The presented case adds to the growing body of literature on COVID-19-associated stroke and underscores the need for a multidisciplinary approach to the management of COVID-19 patients. By sharing this case report, we aim to contribute to the medical knowledge and improve the care of COVID-19 patients, particularly those at risk of developing neurological complications.

## Case presentation

A 68-year-old Caucasian female with a known case of atrial fibrillation (CHA_2_DS_2_-VASc 4), diabetes mellitus type 2, hypercholesterolemia, hypertension, diffuse large B cell lymphoma treated with chemotherapy in 2017(since then patient was on remission), heart failure with a reduced ejection fraction of 39% presented to a tertiary hospital in September 2022, complaining of dry cough for 5 days duration, accompanied by subjective fever, nausea, and dysuria.

Her vital signs were stable upon presentation (blood pressure 114/63 mmHg, breath rate 18/minute, heart rate 73/minute, axillary temperature 36.9 °C). On lung auscultation, the presence of basal crackles was appreciated. Lower limb edema was noted. Neurological exam was grossly unremarkable, she was alert and oriented, power exam was 5/5 for all extremities, Cranial nerves II-XII were intact and speech was normal. The patient reported receiving all three doses of the COVID-19 vaccine (Pfizer-BioNTech), the last dose being in October 2021. The patient's current pharmacological regimen consists of several medications, namely rosuvastatin (10 mg tablet once daily), warfarin (1.5 mg tablet once daily), bisoprolol (7.5 mg tablet once daily), perindopril (5 mg tablet once daily), furosemide (80 mg tablet twice daily), insulin aspart (18 units before breakfast and 10 units before lunch), insulin glargine (24 units once at bedtime), metformin (500 mg tablet twice daily), sitagliptin (100 mg tablet once daily), and ferrous sulfate (190 mg tablet once daily). She had no previous history of surgery and did not smoke or take alcohol or illicit drugs. She reported a history of four pregnancies resulting in the birth of two male and two female offspring without any record of fetal loss. Currently unemployed, she resides with her husband, who provides assistance with daily tasks. Her familial medical history was unremarkable for any cerebrovascular or cardiovascular disorders among first-degree relatives. However, both parents were diagnosed with diabetes.

**Fig. 1:**
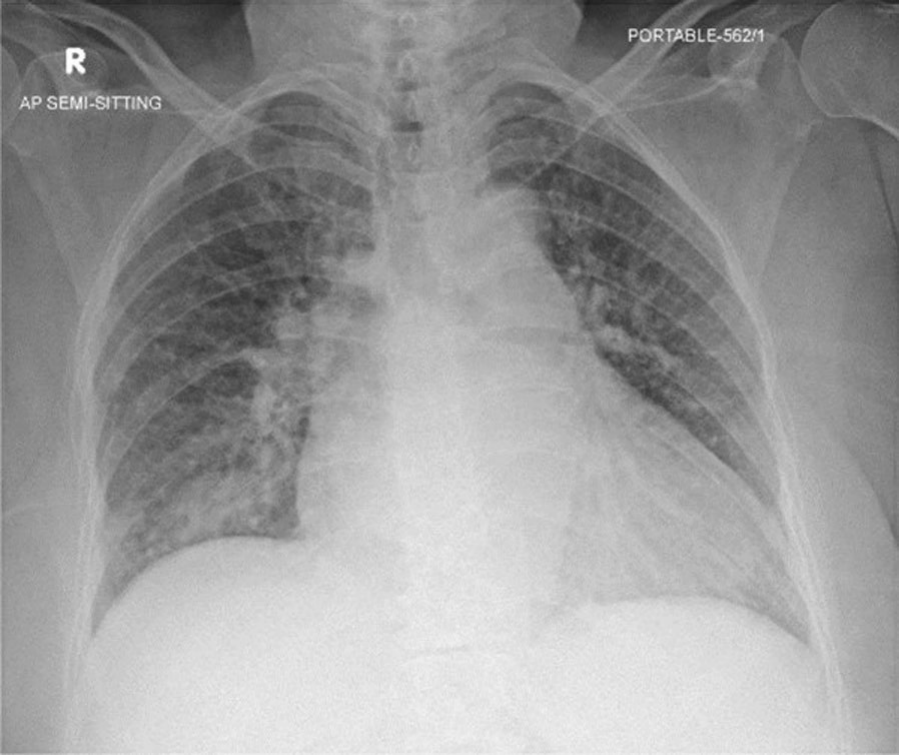
Anteroposterior (AP) chest X-ray on the day of admission

Laboratory investigations revealed the following: hemoglobin 9.4 gm/dL, white blood cells (WBC) 12 × 103 uL, sodium 123 mmol/L, potassium 3.8 mmol/L, magnesium 0.71 mmol/L, C reactive protein (CRP) 239 mg/L, procalcitonin 6.41 ng/mL, creatinine 95 umol/L, international normalized ratio (INR) 3.2, and glucose 12.1 mmol/L. Blood cultures were negative for aerobic and anaerobic organisms. Urinalysis results were positive for WBC 3,672 uL and red blood cells (RBC) 28 uL, and the urine culture was found to be positive for multidrug-resistant Escherichia coli (E. coli) sensitive to meropenem. The COVID-19 rapid antigen test was positive, and the chest X-ray showed bilateral lung congestive changes seen with patchy infiltrate in the right lower lung zone. (Fig. 1).

Based on the investigations done, a diagnosis of COVID-19 with a multidrug-resistant E. coli urinary tract infection was made. The patient was diagnosed with hypervolemic hyponatremia. To manage this condition, a fluid restriction of 1 L per day was implemented, in conjunction with oral administration of 600 mg of sodium chloride tablets three times daily. As per the established protocol for COVID-19 management in patients with multiple comorbidities at our hospital, remdesivir (200 mg IV daily) was initiated for a duration of 5 days. Additionally, meropenem (1000 mg IV three times a day) was administered to address the patient's symptomatic urinary tract infection. This decision was made in light of a previous positive culture for extended-spectrum beta-lactamase (ESBL)-producing E. coli in the patient's urine.

She started to improve clinically and, in terms of laboratory results, planned to be discharged after five days after admission.

On fifth day of admission, the patient developed a sudden onset of confusion and slurred speech. On initial assessment, there was clear dense left-sided hemiplegia with right-sided eyes deviation and a left facial droop. Vitally, the patient maintained saturation in room air with a heart rate of 100 beats per minute, blood pressure of 150/96 mmHg, respiratory rate of 20, and temperature of 36.7 °C. On neurological assessment, she was alert, but disoriented and dysarthric. Cranial nerve examination was significant for left gaze palsy, and testing for power was significant for strength 0/5 on the left side and 4 + /5 on the right side extremities. At that time, the Glasgow Coma Scale (GCS) was 15/15, and the National Institutes of Health Stroke Scale (NIHSS) score was 16. Based on the sudden onset of neurological deficits such as sudden left extremity weakness, left facial palsy and dysarthria, the stroke protocol was activated. The INR was 4.9.

A head CT and perfusion study were performed on after 2 h of initial presentation, which revealed a focal area of matched perfusion defect seen in the right frontoparietal cortical and subcortical region, denoting core infarct with surrounding penumbra and occlusion of the distal anterior M2 segment of the right middle cerebral artery (MCA).

**Fig. 2:**
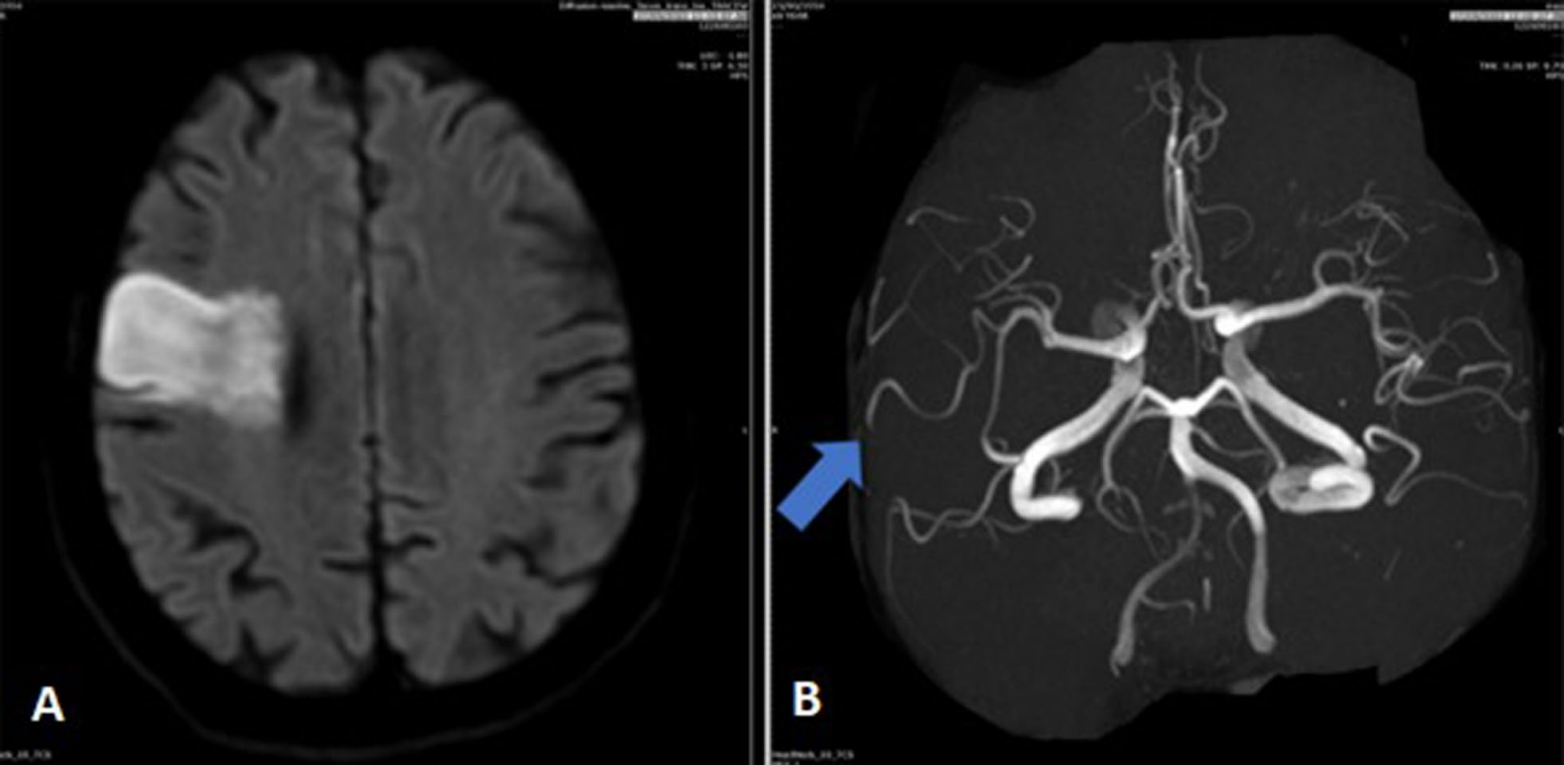
**A** In Diffusion-weighted magnetic resonance of brain imaging showing right frontoparietal diffusion restriction suggesting acute infarction. **B** Magnetic resonance angiography (MRA) of brain demonstrates a paucity of vessels on the right side when compared to the left side and the middle cerebral artery superior trunk occlusion (blue arrow)

A diagnosis of acute ischemic stroke was made, but the patient was not a candidate for thrombolysis or thrombectomy due to an INR of 4.9. One day after the initial CT scan, an additional head MRI and MRA was done which showed right frontal area of 4 × 2.3 cm of diffusion restriction with no hemorrhagic changes (Fig. 2).

Due to swallowing difficulty post-stroke, a nasogastric tube (NGT) was inserted. Interestingly, the patient’s INR increased, reaching a peak of 5.4, despite holding warfarin since admission. However, 6 days later, the patient’s INR went down to 2.2, and she was transferred for rehabilitation.

At follow-up at the rehabilitation institute after one month, the patient still had hemiplegia, and her motor power was tested at 1/5 in the left upper limb and 3/5 in the left lower limb, showing minor improvement. The INR had stabilized in the range of 1.9 to 2.1 during this month. During the six-month follow-up appointment at the Stroke clinic, the patient continued to exhibit residual left-sided weakness, primarily impacting her arm. However, the patient reported no new, recurrent, or worsening neurological symptoms.

## Discussion

We present the case of a 68-year-old woman with confirmed COVID-19, who subsequently suffered from an ischemic cerebral stroke despite receiving anticoagulant therapy.

The association between thromboembolic events and COVID-19 is well known [8, 9]. The incidence of ischemic stroke was ≈10 times higher during the first 14 days after COVID-19 diagnosis as compared with the control intervals [8]. Not only COVID-19 infection itself but also the severity of COVID-19 might lead to increased acute stroke incidence. According to a recent meta-analysis, the total incidence of ischemic strokes was 1.76% among all COVID-19 patients. Based on the severity of the disease, the total stroke rate of patients with severe COVID-19 was 3.37% (95% CI: 1.74–5.44), and that of patients with non-severe COVID-19 was 0.61% [10].

The exact mechanism by which COVID-19 increases the risk of acute stroke remains unknown. Certain studies have proposed the idea that an inflammatory cytokine storm might contribute to a hypercoagulable state and endothelial damage [7]. Moreover, patients with COVID-19 who were admitted to the ICU had higher levels of granulocyte–macrophage colony-stimulating factor, interferon gamma-induced protein 10, monocyte chemoattractant protein-1, macrophage inflammatory protein 1a, and TNF-α, suggesting the presence of this excessive inflammatory process in critically ill patients [9].

Concurrently, other factors that may have contributed to our patient developing stroke could be chronic atrial fibrillation, diabetes and hypertension. The patient had controlled blood pressure readings before and after the stroke event. Even though the electrocardiography (EKG) rhythm was atrial fibrillation rhythm, the transthoracic echocardiogram showed no intracardiac masses or thrombi. At the time of the stroke event, our patient had an INR of 4.9, which would have made an ischemic stroke quite unlikely to happen.

In addition, our patient had hypercholesterolemia, which was controlled by statin use. Hence, previous literature has shown that hypercholesterolemia and hypertension are both independent factors for ischemic stroke [11]. It has been suggested that they have a potential synergistic effect on the risk of ischemic stroke [12]. Both hypercholesterolemia and hypertension contribute to the thickening of carotid artery intima-media, thereby reducing the blood supply to the brain and leading to ischemic stroke [13, 14]. However, the patient was primarily taking rosuvastatin 10 mg; statins control blood lipids, and play a significant role in primary and secondary stroke prevention [15, 16]. Referring to this patient’s INR-controlled lipid panels, it is unlikely to have been the main cause of her ischemic stroke. Studies have shown that individuals with carotid artery stenosis have a higher risk of stroke, particularly ischemic stroke, proportional of the degree of stenosis [17]. However, the CT angiogram in this patient did not reveal any significant stenosis or atherosclerosis in the aortic arch, origin, or cavernous segments of the internal carotid arteries.

Despite anticoagulation, our patient suffered a right MCA infarct. Several questions emerged with this case report that may require further research. First, arterial thromboembolism is rare when compared with venous thromboembolism, specifically as a COVID-19-related sequela [18]. A limited number of cases of arterial thrombotic infarcts due to COVID-19 have been reported in the literature [2, 19–22]. Second, the source of thrombosis in our patient was not confirmed with follow-up studies; thus, we cannot ascertain whether thrombosis was a localized event, an extracranial embolic event, or a combination of both [2, 20]. Finally, our patient was therapeutically anticoagulated with a high INR, yet still had an arterial thrombotic infarct.

This rare presentation presents the question of whether COVID-19 is associated with ischemic stroke despite the supratherapeutic dose of anticoagulants. If yes, then is there a possibility of a future increase in the anticoagulant dose required for similar encounters? Nevertheless, the use of anticoagulation therapy has become a common prophylactic management in COVID-19 due to its ability to trigger a coagulopathy cascade. In our institution, we administer only 40 mg/day of prophylactic enoxaparin to our COVID-19 patients in the inpatient setting as a routine practice (Additional file 1).

## Learning points

1. SARS-CoV-19 infection is associated with an increased risk of thrombotic events.
2. Ischemic stroke may occur in patients with SARS-CoV-19 infection despite therapeutic INR.
3. Caution should be exercised when manipulating doses of anticoagulation therapy in patients with SARS-CoV-19 infection.

## Conclusion

We present a case of middle cerebral artery ischemic stroke in a patient with SARS-CoV-19 infection despite being on warfarin with supratherapeutic INR. In light of this case, it is important for clinicians to be cognizant of the potential for thromboembolic events in COVID-19 patients, such as stroke, even in patients who are receiving therapeutic anticoagulation therapy.

## Supplementary Information

**Additional file 1.** 2013 CARE Checklist is provided as supplementary document.

## Data Availability

Not applicable.
