# Supplementary material for: Ischemic stroke in the setting of supratherapeutic International Normalized Ratio following coronavirus disease 2019 infection: a case report
Source: J Med Case Rep. 2023 May 31;17:223. doi: 10.1186/s13256-023-03936-8 (PMC10229219; doi:10.1186/s13256-023-03936-8)
Supplement: Supplementary file 1 — Additional file 1. 2013 CARE Checklist is provided as supplementary document. [file 13256_2023_3936_MOESM1_ESM.pdf]

CARE Checklist (2013) of information to include when writing a case report

(cc) BY-NC-ND

Topic	Item	Checklist item description	Reported on Page
Title	1	The words "case report" should be in the title along with the area of focus	Line 1
Key Words	2	2 to 5 key words that identify areas covered in this case report.	Line 18-24
Abstract	3a	Introduction—What is unique about this case? What does it add to the medical literature?	Line 12
	3b	The main symptoms of the patient and the important clinical findings	Line 14-15
	3c	The main diagnoses, therapeutics interventions, and outcomes	Line 15
	3d	Conclusion—What are the main "take-away" lessons from this case?	Line 16
Introduction	4	One or two paragraphs summarizing why this case is unique with references	Line 28-31
Patient Information	5a	De-identified demographic information and other patient specific information	Line 36
	5b	Main concerns and symptoms of the patient	Line 37
	5c	Medical, family, and psychosocial history including relevant genetic information (also see timeline).	Line 39-42
	5d	Relevant past interventions and their outcomes	Line 39
Clinical Findings	6	Describe the relevant physical examination (PE) and other significant clinical findings.	Line 38-39
Timeline	7	Important information from the patient's history organized as a timeline	N/A
Diagnostic Assessment	8a	Diagnostic methods (such as PE, laboratory testing, imaging, surveys).	Line 43-45
	8b	Diagnostic challenges (such as access, financial, or cultural)	N/A
	8c	Diagnostic reasoning including other diagnoses considered	Line 55
	8d	Prognostic characteristics (such as staging in oncology) where applicable	N/A
Therapeutic Intervention	9a	Types of intervention (such as pharmacologic, surgical, preventive, self-care)	Line 59
	9b	Administration of intervention (such as dosage, strength, duration)	Line 59
	9c	Changes in intervention (with rationale)	N/A
Follow-up and Outcomes	10a	Clinician and patient-assessed outcomes (when appropriate)	Line 60-61
	10b	Important follow-up diagnostic and other test results	N/A
	10c	Intervention adherence and tolerability (How was this assessed?)	N/A
	10d	Adverse and unanticipated events	N/A
Discussion	11a	Discussion of the strengths and limitations in your approach to this case	N/A
	11b	Discussion of the relevant medical literature.	Line 73-75
	11c	The rationale for conclusions (including assessment of possible causes)	Line 67-72
	11d	The primary "take-away" lessons of this case report	Line 78-81
Patient Perspective	12	When appropriate the patient should share their perspective on the treatments they received	N/A
Informed Consent	13	Did the patient give informed consent? Please provide if requested	Yes ☑ No ☐
